# Lupus mastitis: A rare breast cancer differential diagnosis

**DOI:** 10.1002/ccr3.4416

**Published:** 2021-07-09

**Authors:** Alice Pimentel, Ana Moreira, Sofia Silva, Rita Lages, Joana Noronha

**Affiliations:** ^1^ General Surgery Department Centro Hospitalar do Baixo Vouga Aveiro Portugal

**Keywords:** breast, cancer, mastitis, systemic lupus erythematosus

## Abstract

Lupus mastitis is an uncommon SLE breast manifestation. Clinically, it can present itself as a malignant‐like mass. Therefore, a tissue biopsy is warranted to confirm the diagnosis. The treatment of this condition is pharmacological and directed to the underlying disease. The rarity of this entity demands a high degree of suspicion.

## Graphical abstract

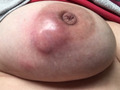

A 35‐year‐old woman presented to the emergency department with a painful right breast mass with 1 month of progressive growth. She had systemic lupus erythematosus (SLE), with renal, hematological, musculoskeletal, and cutaneous involvement and was medicated with hydroxychloroquine 200 mg/day, mycophenolato mofetil 3 gr/day, and prednisolone 7.5 mg/day. On physical examination, she had a swollen hardened breast with a poorly defined mass on the outer lower quadrant (Figure 1). Ultrasound revealed a lobulated vascularized 5 cm mass with liquid areas, suggesting a possible abscess, but not excluding a malignant lesion. The patient was treated with ibuprofen and a course of antibiotics, with subsequent reevaluation 10 days later. Due to progression of inflammatory signs (Figure 2), the mass was drained and biopsied. Histopathology analysis was highly suggestive of breast involvement by SLE—Lupus Mastitis (Figure 3). No malignant features were identified. The patient was referred to the rheumatology department and the dose of hydroxychloroquine and prednisolone was increased to 400 mg/day and 10 mg/day, resulting in significant improvement after 1 month (Figure 4).

**FIGURE 1:**
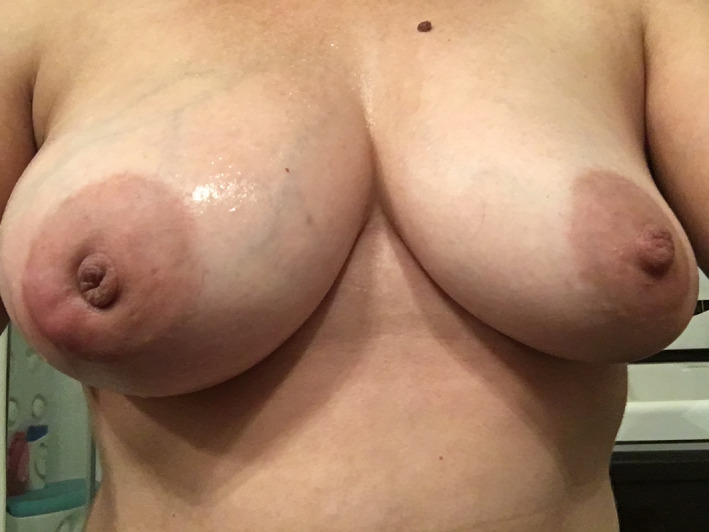
Initial presentation. Swollen hardened right breast with a poorly defined mass on the outer lower quadrant. No palpable lymph nodes were identified

**FIGURE 2:**
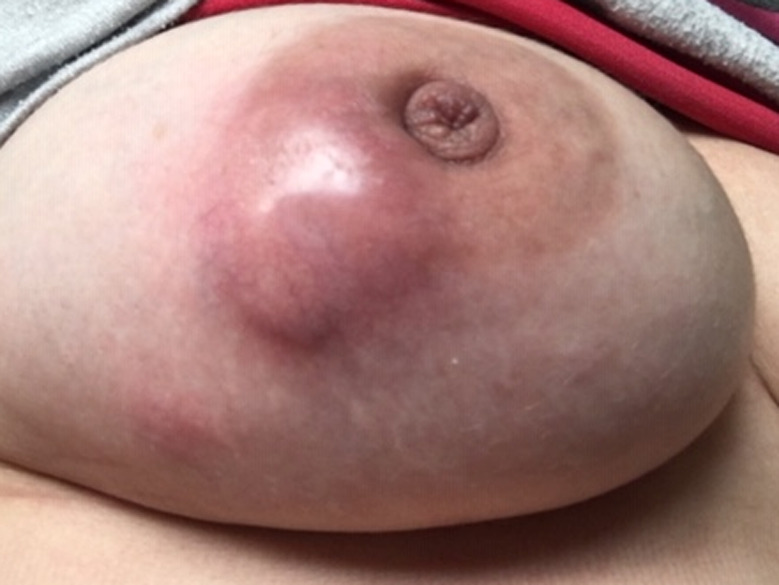
Clinical appearance 10 days after presentation. The mass was drained, and a core‐needle biopsy was performed. Purulent‐like drainage was observed. The fluid cultures were negative

**FIGURE 3:**
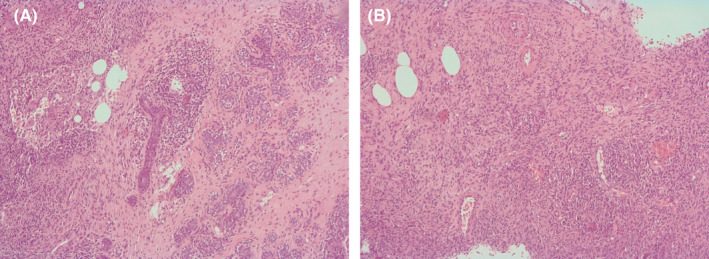
Core‐needle biopsy revealed a fibroinflammatory process: A, perilobular and periductal lymphoid infiltrate; B, Lymphocytic vasculitis. Hematoxylin and eosin stain, magnification x100

**FIGURE 4:**
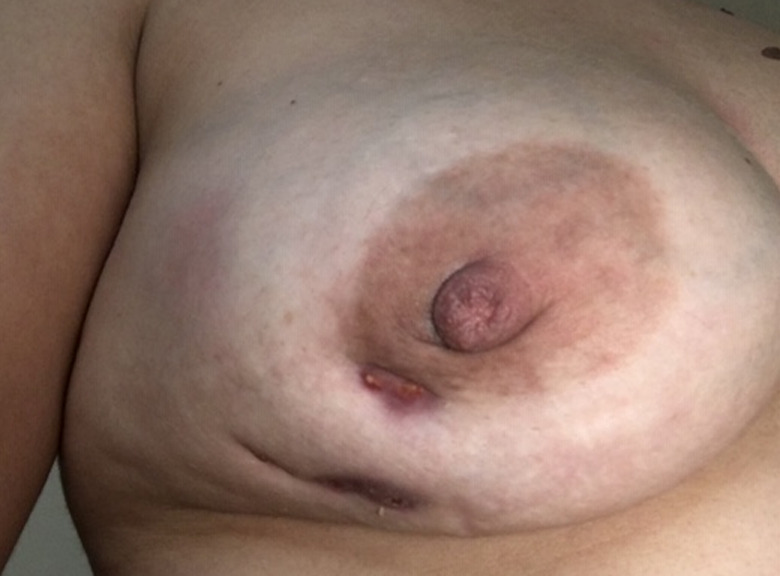
Clinical appearance 1 month after presentation

In case of a breast lesion suggestive of malignancy, patients with SLE should be investigated for lupus mastitis. The diagnosis of this rare condition is histological, and the treatment is directed to the underlying disease.^1^, ^2^

## CONFLICT OF INTEREST

None declared.

## AUTHOR CONTRIBUTIONS

AP: wrote the draft of the manuscript and prepared the figures. AM and SS: involved in writing. RL and JN: revised and approved the final manuscript.

## ETHICAL APPROVAL

All procedures performed were in accordance with the ethical standards of the institutional research committee and with the 1964 Helsinki declaration and its later amendments.

## INFORMED CONSENT

Consent for publication was obtained from the patient.

## Data Availability

All relevant clinical data and images are included in this report. Any additional information is available from the author following reasonable request.

## References

[1] Tanaka Y , Manabe H , Shinzaki W , Hashimoto Y , Komoike Y . A case of lupus mastitis in a patient with systemic lupus erythematosus. Breast J. 2020;26(4):780‐781. 31538694 10.1111/tbj.13582

[2] Rosa M , Mohammadi A . Lupus mastitis: a review. Ann Diagn Pathol. 2013;17(2):230‐233. 23190681 10.1016/j.anndiagpath.2012.09.003

